# Unusual etiology of persistent fever after urinary tract infection: Papillary renal cell carcinoma

**DOI:** 10.1002/jgf2.327

**Published:** 2020-05-13

**Authors:** Yoshinosuke Shimamura, Shuhei Ishikawa, Hideki Takizawa

**Affiliations:** ^1^ Department of Nephrology Teine Keijinkai Medical Center Sapporo Japan; ^2^ Department of Urology Teine Keijinkai Medical Center Sapporo Japan

**Keywords:** computed tomography, papillary renal cell carcinoma, persistent fever, urinary tract infection

## Abstract

This manuscript presents a case report of type 2 papillary renal cell carcinoma presenting with persistent fever and abdominal tenderness after treatment for urinary tract infection. The purpose of this article is to aid physicians in understanding that papillary renal cell carcinomas should be considered in patients with a persistent fever after urinary tract infection and computed tomography was useful to diagnose this entity.

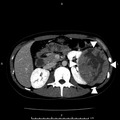

A 23‐year‐old Japanese woman presented with persistence of high fever even after receiving 2 weeks of antibiotic treatment for pyelonephritis. The patient also complained of the left flank pain, anorexia, and malaise, but denied dysuria, urinary frequency, or vaginal discharge. She was not sexually active and a nonsmoker. There was no family history of kidney diseases. Upon physical examination, her respiratory rate was 14 breaths per minute, her heart rate was 115 beats per minute, her blood pressure was 123/84 mm Hg, and her body temperature was 39.4°C. Other notable examination findings were moderate tenderness in her left upper quadrant and left axillary lymphadenopathy. There was no genital ulcer. Blood urea nitrogen level was 7.8 mg/dL, serum creatinine level was 0.62 mg/dL, and urinalysis showed <1 erythrocyte per high‐power field and 1‐4 leukocytes per high‐power field without bacteriuria. Urine culture and blood cultures were negative. Sexually transmitted diseases and recurrent pyelonephritis were less likely based on these findings. Renal abscess and malignant renal tumor were suspected, so that contrast‐enhanced computed tomography was performed, showing a 13 × 10‐centimeter, multiloculated mass with septations on the left kidney (Figure 1) and left axillary lymphadenopathy. Radical ipsilateral nephrectomy with axillary lymphadenectomy was performed. Histopathology of the kidney showed papillae lined by large cells with prominent nucleoli and eosinophilic cytoplasm (Figure 2), and immunohistochemistry had positivity for α‐methylacyl‐CoA racemase and negativity for cytokeratin 7. Besides, left axillary lymph node metastasis was found, but there was no distant organ metastasis. The diagnosis of type 2 papillary renal cell carcinoma (stage IV) was made.

**FIGURE 1:**
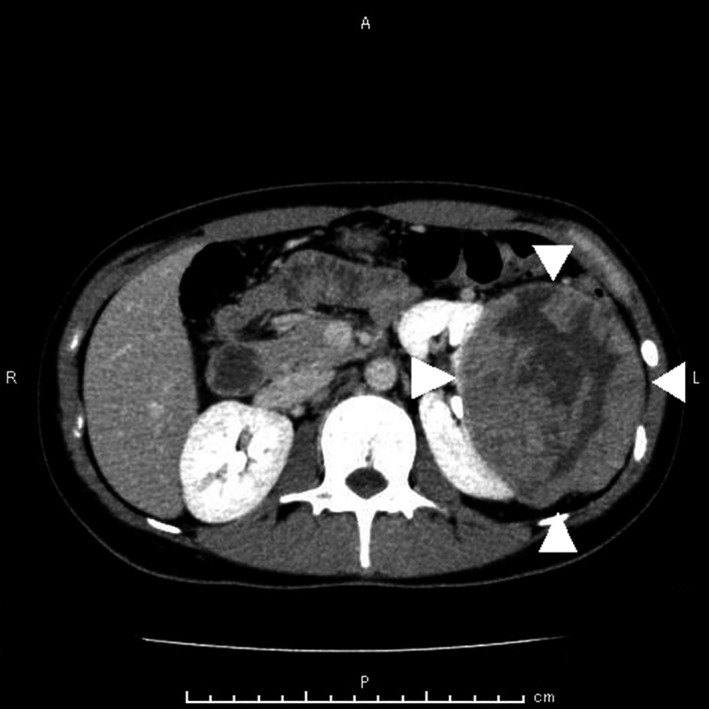
Contrast‐enhanced computed tomography showed a 13 × 10‐centimeter, multiloculated mass with septations on the left kidney (*arrowheads*)

**FIGURE 2:**
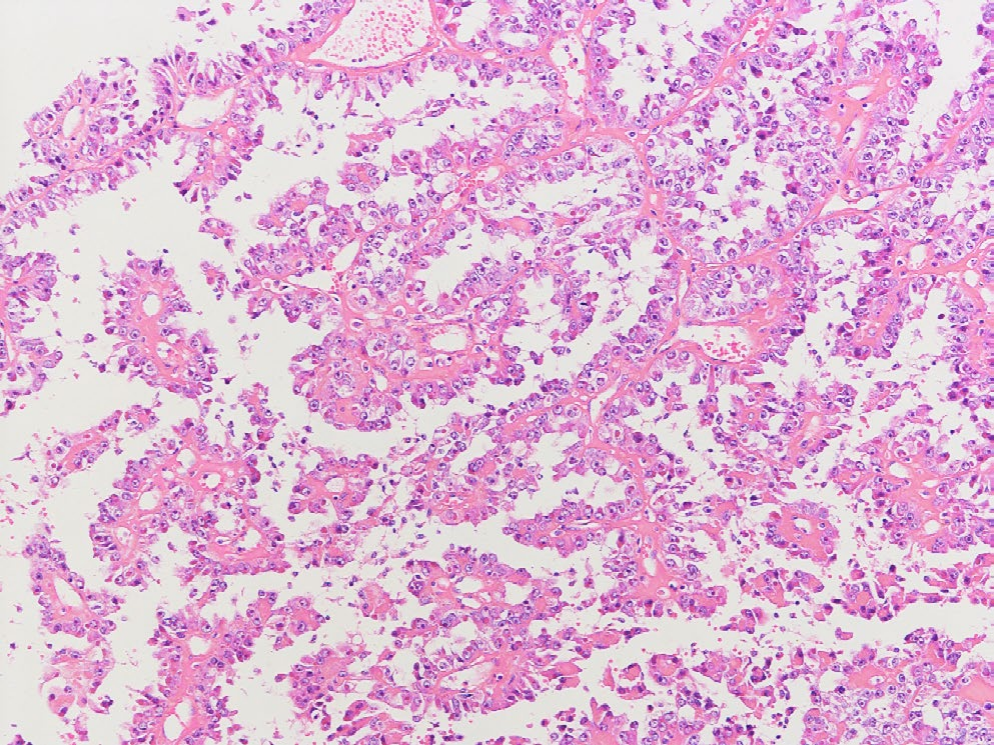
Light microscopy showed papillae configuration lined by large cells with prominent nucleoli and eosinophilic cytoplasm (hematoxylin and eosin, ×400)

Papillary renal cell carcinoma, the second most common form of renal cell carcinomas, is divided into type 1 and type 2. Patients with type 2 often present with more aggressive form and higher rates of metastases than those with type 1, resulting in unfavorable prognosis.^1^ Nephrectomy provides the best chance of cure for localized tumors (stage I to III). By contrast, immune checkpoint inhibitors or anti‐vascular endothelial growth factors are recommended for metastatic tumors (stage IV).^2^, ^3^ The patient has been treating with nivolumab.

Patients with renal cell carcinomas can present with a wide range of symptoms. The classic triad (flank pain, hematuria, and a palpable renal mass) is only seen in nine percent of the patients, whereas fever and anorexia, as in our patient, can be observed in 20 percent of the patients.^4^

Undoubtedly, a high level of suspicion of renal abscess and performing contrast‐enhanced computed tomography is crucial for patients with pyelonephritis who are resistant to antimicrobial therapy;^5^ however, this case illustrates that renal cell carcinoma should also be important to consider in such patients because type 2 papillary renal cell carcinoma, in particular, can present with advanced state at initial encounter.

## ACKNOWLEDGEMENT

None.

## CONFLICT OF INTEREST

The authors have stated explicitly that there are no conflicts of interest in connection with this article.

## ETHICAL APPROVAL

All procedures performed in studies involving human participants were in accordance with the ethical standards of the institutional and/or national research committee at which the studies were conducted (IRB approval number: 2‐019169‐01) and with the 1964 Helsinki declaration and its later amendments or comparable ethical standards.

## INFORMED CONSENT

Informed consent was obtained from all individual participants included in the study.
